# The potential of PARP as a therapeutic target across pediatric solid malignancies

**DOI:** 10.1186/s12885-022-10319-7

**Published:** 2023-04-05

**Authors:** Kaylee M. Keller, Joost Koetsier, Linda Schild, Vicky Amo-Addae, Selma Eising, Kim van den Handel, Kimberley Ober, Bianca Koopmans, Anke Essing, Marlinde L. van den Boogaard, Karin P. S. Langenberg, Natalie Jäger, Marcel Kool, Stefan Pfister, M. Emmy M. Dolman, Jan J. Molenaar, Sander R. van Hooff

**Affiliations:** 1 Princess Máxima Center for Pediatric Oncology, Utrecht, the Netherlands; 2 Hopp Children’s Cancer Center (KiTZ), Heidelberg, Germany; 3 Division of Paediatric Neurooncology, German Cancer Research Center (DKFZ), German Cancer Consortium (DKTK), Heidelberg, Germany; 4 Children’s Cancer Institute, Lowy Cancer Centre, UNSW Sydney, Kensington, NSW Australia; 5 School of Women’s and Children’s Health, Faculty of Medicine, UNSW Sydney, Sydney, NSW Australia; 6 Department of Pharmaceutical Sciences, University Utrecht, Utrecht, the Netherlands

**Keywords:** PARP, Pediatric cancer, Synergy, Replication stress, Ribosomes, DNA damage

## Abstract

**Background:**

Pediatric cancer is the leading cause of disease-related death in children and the need for better therapeutic options remains urgent. Due to the limited number of patients, target and drug development for pediatrics is often supplemented by data from studies focused on adult cancers. Recent evidence shows that pediatric cancers possess different vulnerabilities that should be explored independently from adult cancers.

**Methods:**

Using the publicly available Genomics of Drug Sensitivity in Cancer database, we explore therapeutic targets and biomarkers specific to the pediatric solid malignancies Ewing sarcoma, medulloblastoma, neuroblastoma, osteosarcoma, and rhabdomyosarcoma. Results are validated using cell viability assays and high-throughput drug screens are used to identify synergistic combinations.

**Results:**

Using published drug screening data, PARP is identified as a drug target of interest across multiple different pediatric malignancies. We validate these findings, and we show that efficacy can be improved when combined with conventional chemotherapeutics, namely topoisomerase inhibitors. Additionally, using gene set enrichment analysis, we identify ribosome biogenesis as a potential biomarker for PARP inhibition in pediatric cancer cell lines.

**Conclusion:**

Collectively, our results provide evidence to support the further development of PARP inhibition and the combination with TOP1 inhibition as a therapeutic approach in solid pediatric malignancies. Additionally, we propose ribosome biogenesis as a component to PARP inhibitor sensitivity that should be further investigated to help maximize the potential utility of PARP inhibition and combinations across pediatric solid malignancies.

**Supplementary Information:**

The online version contains supplementary material available at 10.1186/s12885-022-10319-7.

## Background

Worldwide, nearly half a million children aged 0–19 years old will develop cancer each year, and despite technological and treatment advancements over the decades, cancer is the leading cause of disease-related death in children [1]. There remains an unmet need for better therapeutic options for children with cancer, however, the development of mechanism-of-action based therapies is often hindered by the lack of patients, models, and funding for appropriate (pre)clinical investigation. As a result, therapeutic targets and associated biomarkers for pediatrics have often been selected based on vulnerabilities in adult cancers, but investigation into pediatric cancer dependencies suggests that this approach is insufficient [2]. Not only are the pharmacodynamics and adverse drug reactions different in children compared to adults, recent evidence demonstrates that pediatric malignancies are biologically very distinct from adult cancers and have vulnerabilities that should thus be explored independently of adult (pre)clinical data [2–4].

To this end, we sought to explore drug vulnerabilities specific to pediatric solid malignancies, namely Ewing sarcoma (ES), medulloblastoma (MB), neuroblastoma (NB), osteosarcoma (OS) and rhabdomyosarcoma (RMS). While these malignancies all have different etiologies and biology, they are associated with a poor clinical outcome and represent a group of patients in dire need for better therapeutic approaches [5–8]. A drug target effective in cohorts across different malignancy types that have similar targetable vulnerabilities could help overcome the limitations explained above and expedite the development of novel therapeutic approaches that are supported by pediatric-specific preclinical data. Using the publicly available Genomics of Drug Sensitivity in Cancer (GDSC2) dataset, we identify poly (ADP-ribose) polymerase (PARP) as a drug target effective across multiple different pediatric solid malignancies.

PARP is a family of nuclear proteins comprised of 17 members that play an integral role in multiple essential cellular processes, one of which is DNA damage repair. In the presence of single strand DNA breaks, PARP-1 synthesizes the formation of a PAR chain which recruits critical repair proteins to the damage site and facilitates DNA damage repair [9, 10]. Disrupting DNA repair using PARP1/2 inhibitors (here forth referred to as PARP inhibitors) to induce cell death is a well-studied therapeutic approach, particularly for breast cancer [11, 12]. In the presence of PARP inhibitors, single strand DNA breaks cannot be adequately repaired and as such they are converted to double strand DNA breaks. In cells with proficient DNA repair mechanisms, the homologous recombination (HR) repair pathway is activated to ensure genomic integrity and cell survival [9]. However, if cells have deficient double strand break repair mechanisms—such as breast cancers with *BRCA1/2* mutations—PARP inhibition is synthetically lethal and induces cell death. At present, there are four PARP inhibitors approved for the clinical use in adults (olaparib, rucaparib, niraparib and talazoparib), however none have been approved in children yet [13].

In this study, we explore PARP1/2 as a therapeutic target relevant across a wider range of pediatric solid malignancies and we show that efficacy can be improved when combined with conventional chemotherapeutics, specifically topoisomerase inhibitors. Additionally, we propose ribosome biogenesis as a novel treatment biomarker. Altogether, our results demonstrate the potential of PARP inhibitors and combinations in a broader pediatric context and urge further investigation into PARP as a therapeutic target for pediatric patients.

## Methods

### Drug Screening & Expression Datasets

In this study, we use the Genomics of Drug Sensitivity in Cancer version 2 (GDSC2) database to identify potential therapeutic targets in pediatric cancer cell lines [14]. This database includes drug sensitivity data of a 198-compound drug library that has been tested on a range of both pediatric and adult cancer cell lines (44–808; median = 742). Cell viability was determined following 72-hour drug incubation and the half maximal concentration that inhibits viability (IC_50_) and area under the curve (AUC) values are reported. Additionally, this database includes genomic and expression data of the cell lines which we also used in our study.

### Cell lines & culture

A panel of 32 cell lines was used in this study and included six ES cell lines (EW7, Sim (EW24), RD-ES, A-673, POE and sta-et-1), five MB cell lines (Daoy, D283-med, D341-med, UW228.2, and Med-Meb-8a), seven NB cell lines (IMR32, SJ-NB-6, SK-N-BE, SJ-NB-8, NGP, SK-N-AS, and SH-SY5Y), seven OS cell lines (U-2OS, Saos-2, Ior-os-9, MG-63, HOS, Ior-os-14 and Ior-os-18) and six RMS cell lines (RMS-1, RH-30, RD, RMS-YM, Rh18, Rh41). All cell lines used in this study were obtained from the American Type Culture Collection or via historic collaborations and the identity of each cell line was validated by short tandem repeat (STR) analysis. Cells were cultured in appropriate medium with supplementation (outlined in Supplementary Table 1), grown at 37 °C and 5% CO_2_ and regularly tested for mycoplasma infection.

### Cell viability assay

Cells were seeded in black 384-well plates (Corning, 3764) according to predetermined densities based on cell growth rate of each cell line (250–10,000 cells per well). Cells were cultured for 16–24 h under standard culturing conditions (37 °C, 5% CO_2_) before being treated with compounds for either high-throughput drug screens or validation screens. Following 72-hour incubation with the compounds at standard culture conditions, cell viability was measured using the 3-(4,5-dimethylthiazol-2yl)-2,5-diphenyltetrazolium (MTT) assay [15].

### Compound screening

High-throughput drug screens were conducted in collaboration with the high-throughput screening facility of the Princess Máxima Center [16]. The screens were performed with 384-well plates and a library containing 198 drugs using the high-throughput screening facility (Beckman Coulter with a Biomek i7 Automated Workstation). Using the Echo 550 dispenser, the drugs (in DMSO or MQ, at different concentrations) were added to the wells containing the cells, at final concentrations of 0.1 nM, 1 nM, 10 nM, 100 nM, 1 μM and 10 μM (0.25% DMSO or MQ). Combination validation screens were conducted using a 10 × 10 matrix of five-fold concentration ranges from 0.03 nM to 10 μM (0.25% DMSO) using the D300e Digital Dispenser (TECAN). For all screens, cells treated with DMSO were used as positive controls and for the high-throughput screens, cells treated with staurosporine (final concentration of 10 μM) were used as negative controls. Supplementary Table 2 summarizes the compounds included in this study.

### Data Processing & Statistics

For the high-throughput screens, IC_50_ and AUC values were derived from the dose-response curves using the package *drc* in the R statistical environment (version 4.2.0) [17]. For validation screens, IC_50_ and AUC values were derived from the dose-response curves using GraphPad Prism version 9.0 for windows. For all screens, the data was normalized to the DMSO-treated cells (defined as 100% viability) and empty controls (defined as 0% viability). IC_50_ values at 72-hours were calculated by determining the concentrations of the drug needed to achieve a 50% reduction in cell viability. AUC values were calculated by determining the definite integral of the curve.

AUC values of the GDSC2 dataset were used to explore differential compound sensitivities of pediatric versus adult cell lines by adapting gene set enrichment analysis (GSEA; using compound sets instead of genes). For this we compared AUC values of pediatric and adult cancer cell lines for all 198 compounds using the limma R package and used the resulting t statistics as input for the fgsea R package. We grouped the compounds based on their annotated target or target pathway (analogous to gene sets) as additional input for the GSEA analysis. In addition, we used GSEA to determine which genesets (KEGG subset from MSigDB version 7.5.1) [18] were correlated with talazoparib sensitivity (AUC) in pediatric cell lines. For this analysis, genes were ranked based on the t statistic calculated using limma with the talazoparib AUC values as a continuous and the tumor type as a categorical variable.

## Results

### PARP as a drug target in pediatric solid malignancies

**Fig. 1:**
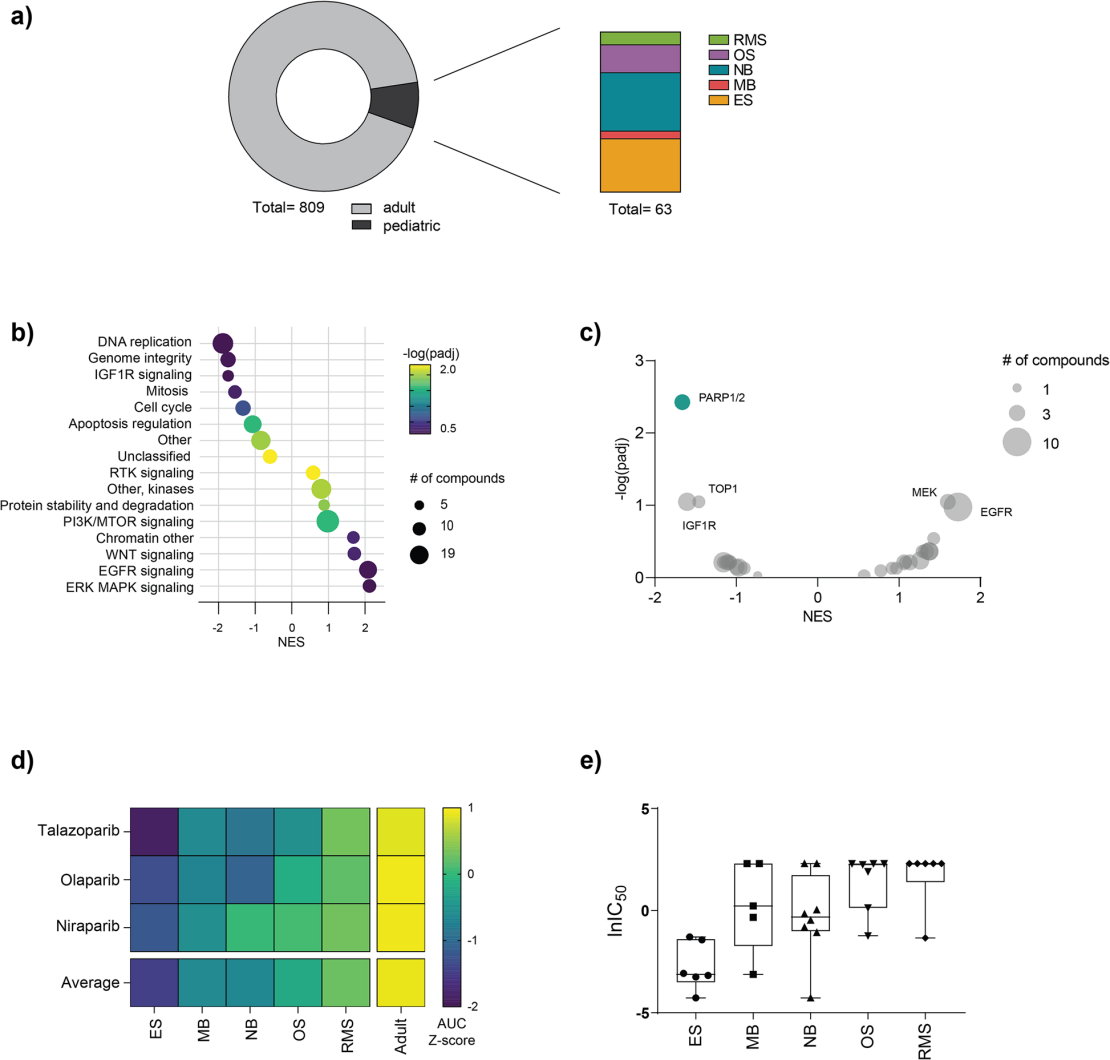
Pediatric cancer cell lines are overall sensitive to PARP inhibition. **a** Overview of the cell lines and pediatric malignancy types represented in the GDSC2 dataset. **b** Normalized enrichment scores (NES) of compounds grouped according to biological pathways (at least five members) comparing pediatric versus adult cancer cell lines. The size of each dot represents the number of compounds targeting that pathway and the color represents the -log (padj). Negative NES values indicate more sensitivity in pediatric cell lines. **c** Volcano plot of differential sensitivities in pediatric versus adult cancer cell lines. Each dot represents a different drug target, and the size represents the number of compounds included. **d** Heatmap representing the AUC Z-scores for three different PARP inhibitors (talazoparib, olaparib and niraparib) in pediatric (ES, MB, NB, OS and RMS) and adult cell lines included in the GDSC2 dataset. **e** Box plot of lnIC_50_ values for 32 different pediatric cancer cell lines following 72-hour treatment with talazoparib

Using the publicly available GDSC2 database, we investigated differential drug sensitivity of pediatric versus adult cancer cell lines (Fig. 1a). Following statistical analysis of biological pathways being targeted by the drugs in the GDSC2 library, we observed that in comparison to adult cancer cell lines, pediatric cancer cell lines are generally more sensitive to compounds targeting DNA replication, genome integrity and IGFR1 signaling but less sensitive to targeting of ERK, MAPK, EGFR and WNT signaling pathways (Fig. 1b). Our findings mirror recently published results where DNA replication and IGF1R signaling were identified as key dependencies unique to pediatric tumor types, further suggesting that drug targets within these pathways could be useful therapeutic approaches in pediatric cancer [2, 19].

To elucidate which targets in these pathways are of particular interest, we looked for specific compounds to which pediatric cancer cell lines were more sensitive (versus adult cancer cell lines) and found that PARP inhibitors (talazoparib, niraparib and olaparib) demonstrated the greatest and most significant efficacy in pediatric cancer cell lines (Fig. 1c). When looking at the efficacies of these three PARP inhibitors in specific pediatric (ES (*n* = 19), NB (*n* = 23), MB (*n* = 4), RMS (*n* = 7) and OS (*n* = 10)) and adult (*n* = 686) tumor cell lines, we observed a range of sensitivities. Within the pediatric cell lines, Ewing’s sarcoma was the most sensitive and rhabdomyosarcoma was the least sensitive and overall, the pediatric tumor types had lower AUC Z-scores compared to adult cancer cell lines (Fig. 1d).

We next validated the observed sensitivity across pediatric tumor types in the GDSC2 dataset, by screening an in-house panel of 32 pediatric cancer cell lines (summarized in Supplementary Table 1) with the PARP inhibitors talazoparib and olaparib. Consistent with the drug responses in the GDSC2 dataset, we observed that Ewing’s sarcoma cell lines demonstrated the greatest sensitivity to PARP inhibition (average IC_50_ = 0.12 μM), and rhabdomyosarcoma and osteosarcoma cell lines were the least sensitive overall (average IC_50_ = 3.03 μM and 3.74 μM, respectively; Fig. 1e). Additionally, we also observed greater in vitro sensitivity to talazoparib compared to olaparib (Supplementary Fig. 1). Altogether, our findings were consistent with the GDSC2 dataset and other published literature which demonstrates differential sensitivity to PARP inhibitors between and within pediatric tumor types [20].

### High-throughput combination screening

The clinical use of single compound treatment with PARP inhibitors has been greatly challenged by both inherent and developed resistance to PARP inhibition. An increase in drug efflux, restoration of HR, and stabilization of stalled replication forks are some of the resistance mechanisms observed [11, 12]. As such, we explored compounds that could be combined with PARP inhibitors to improve efficacy by performing high-throughput combination screens on one cell line of each pediatric entity (A673, D341-med, HOS, NGP and RMS-YM) that demonstrated sensitivity to talazoparib alone (IC_50_ < 0.5 μM; Supplementary Fig. 2a). Using three anchor concentrations of talazoparib (IC_15_, IC_25_ and IC_50_ for each cell line) combined with a drug library containing a 6-fold concentration range of 198 compounds that are in (pre)clinical development for children, we generated a robust dataset containing nearly twenty thousand combinations in total (Supplementary Fig. 2b). It has been previously shown that combination therapy can improve potency, overall efficacy or both and that multiple metrics should be considered to most robustly select potential combinations [21]. To this end, we performed a similar method and calculated three outcome metrics for each combination: 1) fold change in maximum effect at the highest library drug concentration (∆E_max_), 2) fold change in IC_50_ value (∆IC_50_) and 3) average synergy score according to the bliss independence model [22].

**Fig. 2:**
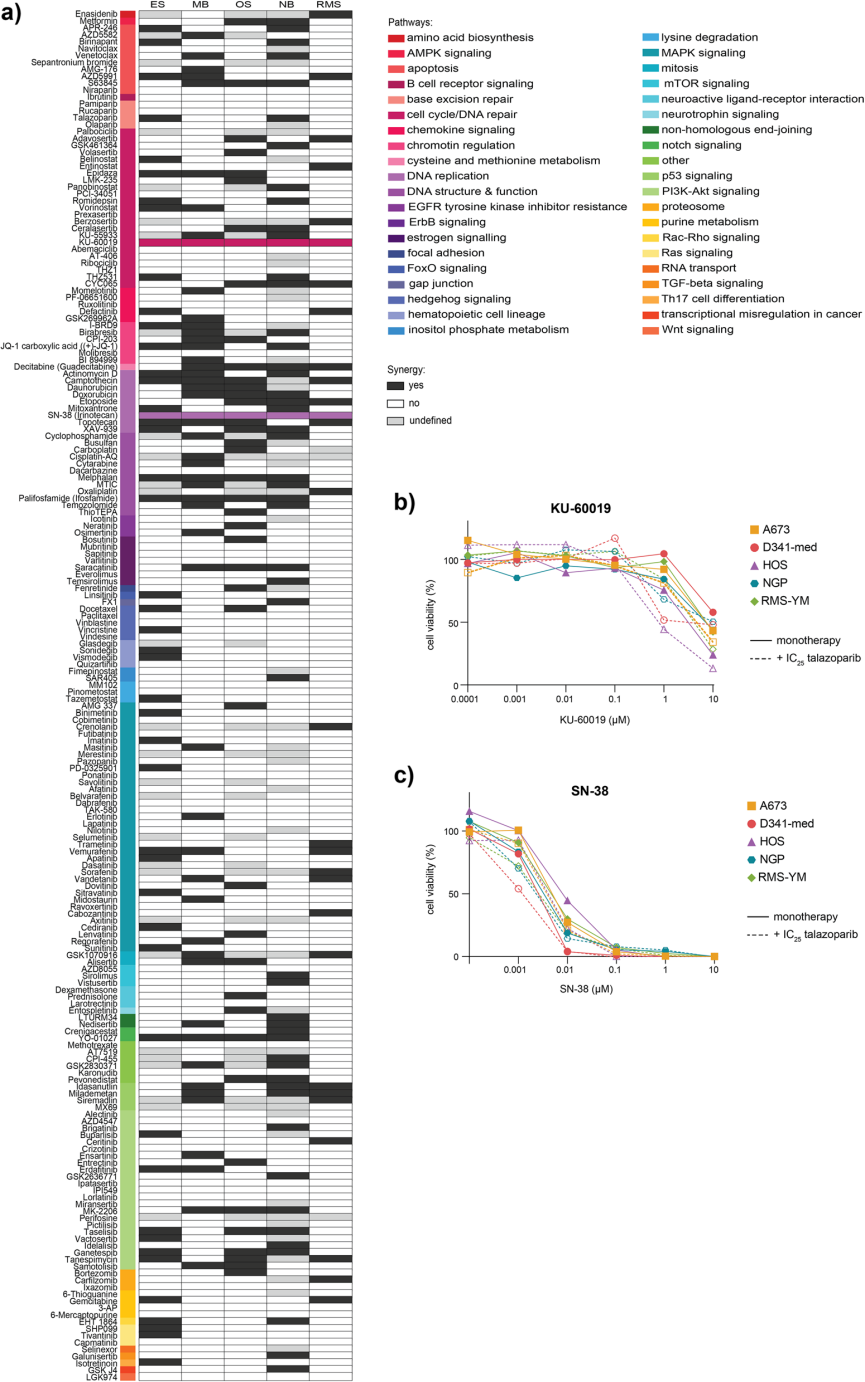
High-throughput drug screening demonstrates increased synergy with compounds targeting DNA replication and chromatin regulation pathways. **a** Summary heatmap of high-throughput screening combining a drug library with three anchor concentrations of talazoparib in ES (A673), MB (D341-Med), OS (HOS), NB (NGP) and RMS (RMS-YM) cell lines. Compounds were considered synergistic (black or colored in the case of KU-60019 and SN-38) if they demonstrated improved efficacy in the average ∆IC_50_, ∆E_max_ or average bliss score. If a compound did not induce an improvement in at least one of the metrics, it was not considered synergistic (white) and light grey boxes represent compounds that were not screened. Colored bars indicate the biological pathway targeted by each compound listed. **b-c** Dose response curves for KU-60019 and SN-38 monotherapy (solid line) or in combination with the IC_25_ of talazoparib for each cell line (dashed line). Curves are representative of a single biological replicate

To evaluate potential synergistic combinations, we used these metrics averaged across the three anchor concentrations of talazoparib. As this step of our analysis was intended to identify potential candidates, combinations that yielded an average fold change greater than five in E_max_ or IC_50_ or had an average bliss score greater than zero were considered. Overall, 47, 50, 43, 52 and 28 of the tested combinations were regarded synergistic in at least one of the three averaged metrics in ES, MB, OS, NB and RMS, respectively. Despite finding numerous synergistic hits in each malignancy type, there was a striking lack of overlapping synergy and only two combinations yielded synergy across the five different pediatric tumor types included in our study: talazoparib combined with KU-60019 or SN-38 (Fig. 2a).

The compound KU-60019 is a small molecule inhibitor of Ataxia-Telangiectasia Mutated (ATM) and following combination treatment with talazoparib, we observed synergy as evidenced by shifts in dose-response curves (Fig. 2b). However, despite the modest improvement in IC_50_ values, the maximum effect of the combination remained limited, indicating that higher concentrations would likely be necessary to induce complete cell killing (Supplementary Fig. 3). At in vitro drug concentrations greater than 10 μM, the clinical applicability of a compound becomes questionable and as such, we did not further explore the potential of PARP-ATM inhibition as a combination across different pediatric tumor types.

Talazoparib combined with SN38 (the active metabolite of irinotecan, a TOP1 inhibitor), on the other hand, demonstrated very promising effects. Having an IC_50_ range of 2–8.5 nM, the cell lines were very sensitive to SN-38 alone. However, with the addition of talazoparib, greater efficacy was observed and the IC_50_ range was reduced to 1.1–4.1 nM (Fig. 2c; Supplementary Fig. 4). Altogether, combined TOP1 and PARP inhibition demonstrated synergy and could induce maximum cell killing at low concentrations of both drugs, marking it as an interesting synergistic candidate to be explored further.

### Combined PARP and TOP1 treatment is synergistic in PARP inhibitor sensitive cell lines

**Fig. 3:**
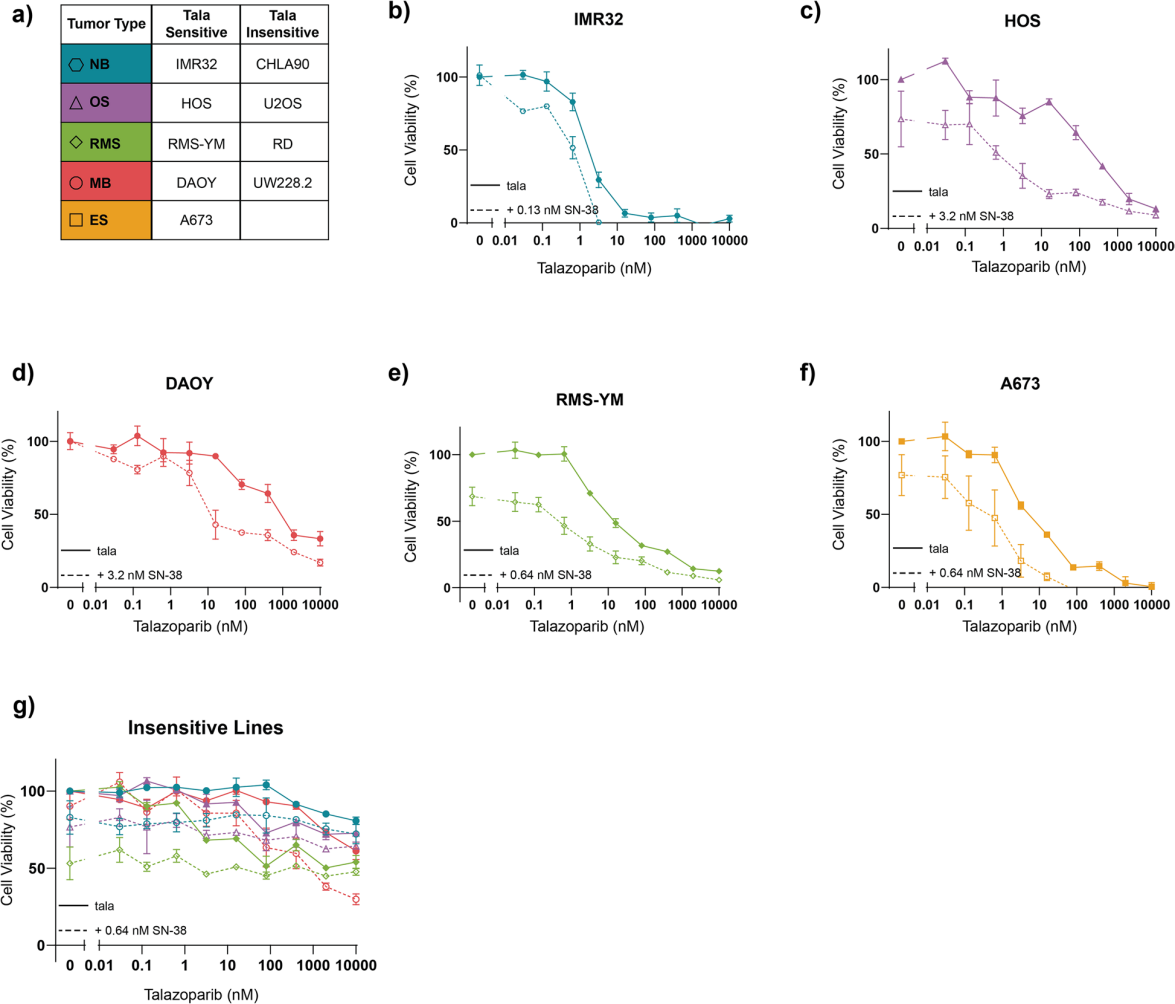
Combined PARP and TOP1 inhibition is synergistic in talazoparib sensitive cell lines. **a** A summary of cell lines used in this study. **b-g** Dose-response curves following 72-hour treatment with talazoarpib monotherapy or in combination with SN-38. In all curves, the color and shape of data points indicate the tumor type. Solid lines represent talazoparib monotherapy and dashed lines represent combination therapy. All curves represent the average of two replicates and error bars indicate the standard error of the mean (SEM)

To investigate our findings further, we conducted combination drug screens using a wider range of concentrations on a panel of pediatric cancer cell lines of each malignancy type which included talazoparib sensitive (A673, IMR32, RMS-YM, HOS and DAOY) and insensitive cell lines (CHLA90, RD, U2OS and UW228.2; summarized in Fig. 3a). Talazoparib insensitive cell lines were defined as those whose measured viability was greater than 50% following 72-hour treatment with 10 μM talazoparib only. Due to the sensitivity of Ewing’s sarcoma to PARP inhibition in general, our panel did not include an insensitive Ewing’s sarcoma cell line.

Consistent with our high-throughput screening results, we observed improved efficacy when SN-38 was combined with talazoparib in sensitive cell lines. Although different concentrations were required depending on the tumor type, all cell lines demonstrated synergy with the addition of 0.13–3.2 nM of SN-38. The NB cell line IMR32, which has a homozygous mutation of the DNA damage repair protein ataxia telangiectasia mutated (ATM), was the most sensitive to talazoparib treatment alone (IC_50_ = 1.79 nM) and it demonstrated synergy with the addition of 0.13 nM of SN-38 (IC_50_ = 0.61 nM; Fig. 3b) [23]. In the OS and MB cell lines (HOS and DAOY, respectively), 0.13 nM of SN-38 was insufficient to induce strong synergistic effects. For these cell lines a greater concentration of SN-38 (3.2 nM) was required to significantly improve efficacy, which resulted in a 40-fold and 300-fold decrease in IC_50_ for DAOY (IC_50_ = 19.8 nM) and HOS (IC_50_ = 0.89 nM), respectively (Fig. 3c-d). The remaining two tumor types (RMS and ES) also demonstrated synergy and strong synergistic effects were observed with the addition of 0.64 nM of SN-38 in the cell lines RMS-YM and A673 (Fig. 3e-f). In contrast, the talazoparib insensitive cell lines were resistant to the addition of SN-38, regardless of the concentration (Fig. 3g; Supplementary Fig. 5).

**Fig. 4:**
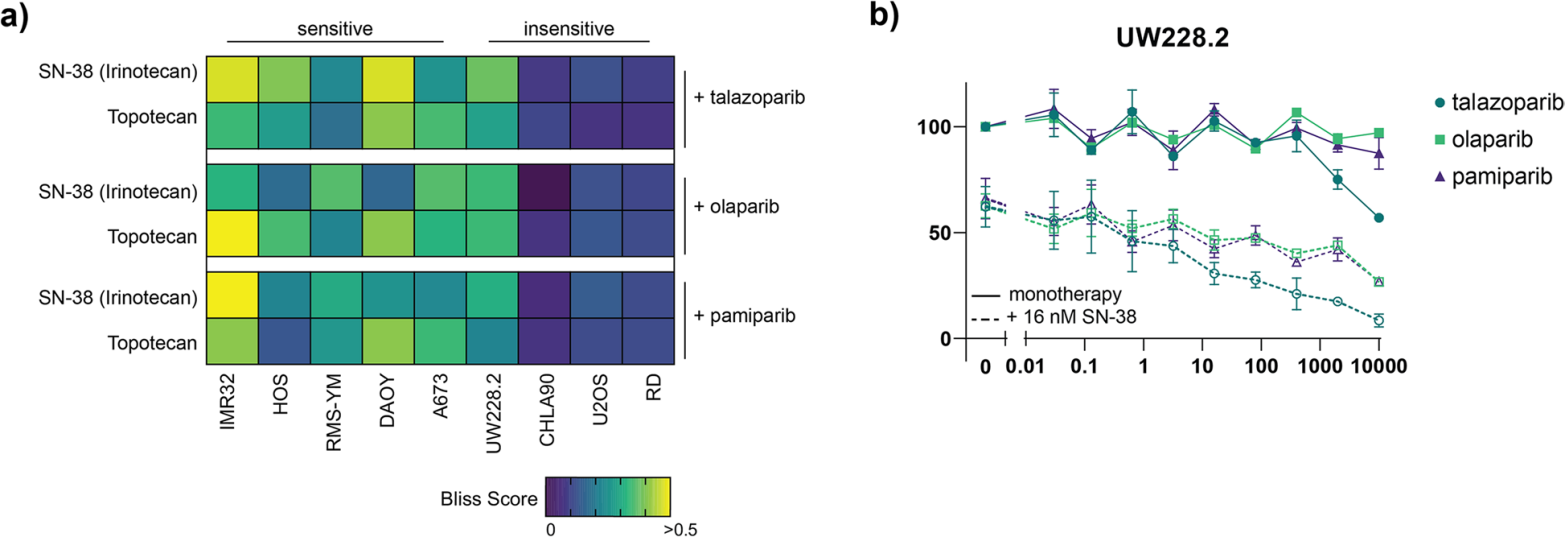
Maximum effective synergy of combined PARP and TOP1 inhibition is not inhibitor specific. **a** Heatmap indicating the maximum effective bliss synergy score for TOP1 inhibitors (SN-38 and topotecan) combined with three different PARP inhibitors (talazoparib, olaparib and pamiparib) in a panel of pediatric cancer cell lines. **b)** Dose-response curves for the MB cell line UW228.2 following 72-hour incubation with PARP inhibitors only (solid lines) or combined with 16 nM SN-38 (dashed lines). All curves represent the average of two replicates and error bares indicate the standard error of the mean (SEM)

Altogether our results show that talazoparib is most synergistic with SN-38 in cell lines that are already sensitive to talazoparib treatment. To investigate whether this was an effect specific to talazoparib, we further screened our panel of cell lines with two other PARP inhibitors: olaparib and pamiparib (BGB-290). These PARP inhibitors do not possess the same PARP-entrapment properties as talazoparib and had higher IC_50_ values when used as monotherapy in vitro (Supplementary Figs. 6 and 7) [24]. Additionally, we combined all three PARP inhibitors with another TOP1 inhibitor, topotecan, to further investigate whether observed effects were inherent to SN-38 treatment (Supplementary Figs. 8–10). Interestingly, despite differences in monotherapy efficacy, very similar synergistic effects as previously noted with SN-38 were observed when the PARP inhibitors were combined with topotecan. Again, we observed higher maximum effective synergy scores (defined as the highest bliss independence score that is associated with a cell viability < 50%) in the sensitive cell lines than in the insensitive cell lines (Fig. 4a). The exception is the insensitive MB cell line (UW228.2) where maximum effective synergy scores indicate some improved efficacy of combination treatment, but only at therapeutically irrelevant talazoparib concentrations (Fig. 4b).

### Potential biomarkers for PARP inhibition in pediatric solid malignancies

As evidenced by our study and other published results demonstrating differential sensitivity to PARP inhibitors and combinations, there exists a need for treatment biomarkers [20]. To explore potential mechanisms underlying PARP inhibitor sensitivity in pediatric solid tumors, we examined biological pathways and genes correlated with talazoparib sensitivity in the pediatric cancer cell lines included in the GDSC2 database [25] .

**Fig. 5:**
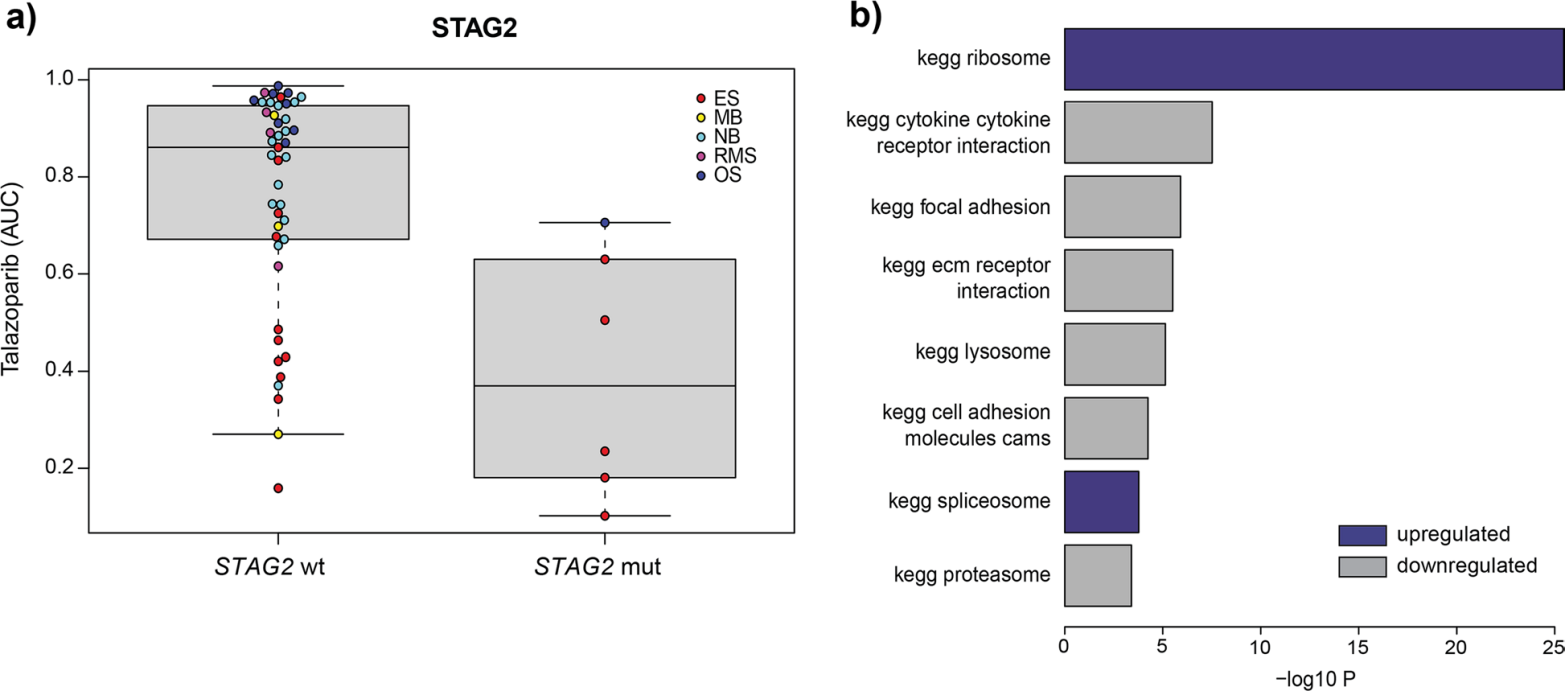
Potential biomarkers for PARP inhibitor sensitivity. **a** Boxplot of AUC values following talazoparib treatment for pediatric cancer cell lines in the GDSC2 dataset with and without *STAG2* mutation. **b** GSEA showing biological pathways (KEGG) that are positively (blue) or negatively (grey) associated with talazoparib sensitivity in pediatric cancer cell lines included in the GDSC2 dataset (adjusted *p* value < − 0.001)

As was found in previous studies, *STAG2* mutations correlated with sensitivity to PARP inhibition with talazoparib, particularly in Ewing’s sarcoma (Fig. 5a). While *STAG2* mutations are part of the genomic landscape of Ewing’s sarcoma and have been implicated in sensitivity to PARP inhibitors, it is not a mutation that is frequently observed in other pediatric cancer types and therefore does not explain or predict the sensitivity observed in Ewing’s sarcoma and other pediatric cell lines lacking this mutation [26, 27].

Therefore, we also performed a GSEA analysis to identify gene expression patterns associated with talazoparib sensitivity for all pediatric cancer cell lines tested. There we observed a highly significant correlation between talazoparib sensitivity and upregulated expression of the KEGG ribosomal pathway (Fig. 5b). Interestingly, in a recent study, PARP inhibition has been implicated in the dysregulation of nucleolar stress management, suggesting a potential mechanism linking the regulation of ribosomal genes with talazoparib sensitivity [28, 29]. Also of note is that the link observed in pediatric lines is also present in adult cell lines (albeit somewhat less pronounced), suggesting this association is more broadly applicable (Supplementary Fig. 11). Although outside the scope of our study, to fully investigate this novel biomarker, it would be beneficial to expand our mechanistic understanding of PARP inhibitor effects on ribosomal pathways. A first step could be to elucidate differences in rRNA production and nucleolar organization of key ribosomal proteins such as RPL5 and RPL11 in PARP inhibitor sensitive and insensitive cell lines.

## Discussion

In our study, we use publicly available datasets to identify PARP as a potential drug target for pediatric solid malignancies (OS, ES, NB, MB and RMS). Being that pediatric cancers account for a very small proportion of cancer diagnoses worldwide, there is a certain degree of hesitancy to fund pediatric-specific (pre)clinical drug development and the execution of robust clinical trials remains difficult. The existence of a drug target across different malignancy types could help mitigate these challenges and aid in the ethical development of safe therapeutics for children with cancer. Furthermore, the poor prognostic outlook for patients with relapsed solid tumors urges the development of more targeted treatment approaches, such as PARP inhibition. Currently, there are no clinically approved PARP inhibitors for children, however the results of a recently published phase 1 clinical study evaluating olaparib in pediatric patients with refractory solid tumors were promising and there are multiple ongoing clinical studies to evaluate the safety, dosing and efficacy of PARP inhibitors in children (NCT02392793, NCT04544995, NCT01858168, among others) [30].

Our study is not the first to identify PARP as a potential target in pediatric solid malignancies. In fact, in our recent systematic review of replication stress as a therapeutic target for pediatric cancers, PARP presented as the most robustly investigated target within these pathways [20]. However, despite an abundance of data, PARP has not been thoroughly considered in the broader context of pediatric cancer. Therefore, we used a combination of publicly available and self-generated data to explore the potential of PARP more thoroughly as a target across different malignancy types. Unsurprisingly, we observed intra- and inter-tumoral differential sensitivity to PARP inhibition in pediatric cancer cell lines, highlighting the necessity for both novel combinations to improve efficacy and biomarkers to select sensitive phenotypes.

Following extensive high-throughput drug screens using a library of compounds that are in (pre)clinical development for pediatrics, we found numerous potential synergistic combinations within each of the different malignancy types, including doxorubicin and cyclophosphamide which have previously demonstrated synergy when combined with PARP inhibition in some pediatric tumor types [10]. However, there were only two combinations that demonstrated synergy across all the different tumor types included in our study. Further investigation eventually revealed only one true candidate combination: talazoparib and SN-38. As talazoparib is known for its superior PARP-entrapment properties we hypothesized that this could possibly be driving improved synergistic efficacy. If PARP becomes trapped on the DNA, it prevents appropriate progression of the replication fork and initiates additional DNA damage and ultimately cell death. Combined with the genotoxic effects of TOP1 inhibition, it is possible that combination treatment increases DNA damage to a rate at which cells cannot cope. After testing the combination using two other PARP inhibitors with slightly different mechanisms of action than talazoparib, we observed similar maximum effective synergy scores. This suggests that the observed effects are likely an effect of PARP inhibition itself and that the mechanism of synergy is not fully dependent on DNA damage induced by PARP-entrapment.

One possible mechanism that could be underlying the observed synergism between PARP and TOP1 inhibitors is related to ribosomes. In a recent study, it was demonstrated that TOP1 inhibition combined with a ribosomal gene transcription inhibitor caused global replication stress independent of DNA damage and thereby induced cell growth inhibition [31]. PARP itself has also recently been linked to ribosomal biogenesis, which is a process that is generally upregulated in cancer cells to promote cell growth [29, 32]. In this study, Kim et al. were able to show that inhibition of PARP induced cell growth inhibition via a mechanism independent of DNA damage repair. This mechanism has been further elucidated in another study where the effects of olaparib on rRNA biosynthesis and p53 activation were investigated [28].

In conjunction with the strong correlation we observed between PARP inhibitor sensitivity and ribosomal pathways, we believe this is a mechanism that should be explored further, especially in the context of treatment biomarkers [33, 34]. In our study, we not only observed differential sensitivity between and within tumor types to PARP inhibition alone, but our investigation into combined TOP1 and PARP inhibition revealed a similar diversity in response. Historically, sensitivity to PARP inhibitors has been attributed to HR repair deficiency by way of *BRCA1/2* mutations [35–37]. However, this is not a commonly observed mutation in pediatric tumors [25]. In the pediatric setting, it is likely that PARP inhibitors are synthetically lethal via different DNA repair deficiencies such as *STAG2* mutations. STAG2 is a component of the cohesion complex which is essential to multiple cellular functions, including HR repair, and multiple studies have suggested it as a biomarker for PARP inhibition [26, 27, 38, 39]. Additionally, the expression of the EWS-FLI1 gene fusion has been proposed as a biomarker for PARP inhibition in Ewing’s sarcoma. This fusion gene is observed in approximately 85% of all Ewing’s sarcoma and has been previously shown to induce DNA damage, which can be potentiated with the inhibition of PARP [40–43]. Both *STAG2* mutations and the expression of EWS-FLI1 fusion are frequently encountered in Ewing’s sarcoma and are likely an explanation to why this tumor type demonstrates an overall greater sensitivity to PARP inhibition compared to other pediatric tumor types. However, in the broader pediatric context, this is an incomplete explanation. In our study, we show that pediatric cancer tumor types other than Ewing’s sarcoma also demonstrate sensitivity to PARP inhibition, independent of *STAG2* mutation or EWS-FLI1 fusion. Altogether, our study encourages further investigation of alternative biomarkers for PARP inhibition, such as ribosomal pathways.

## Conclusion

Collectively, our study highlights the potential of PARP as a therapeutic target across different pediatric tumor types and suggests combination with TOP1 inhibition to improve efficacy. However, the understanding of the mechanisms driving PARP inhibitor sensitivity and associated biomarkers remain incomplete. Being supported by decades of literature, it is clear that DNA damage repair deficiencies are a driver of PARP inhibitor sensitivity, but certainly considered in the context of pediatric cancers, this is evidently an incomplete explanation. In our study, we propose ribosome biogenesis as an additional component to PARP inhibitor sensitivity that should be further investigated to help broaden utility of PARP inhibition and combinations across pediatric solid malignancies.

## Supplementary Information

**Additional file 1: Supplementary Table 1.** Summary of cell lines, medium and supplements used in this study. **Supplementary Table 2.** Compounds and associated targets included in the hight-throughput drug library.

**Additional file 2: Supplementary Fig. 1.** Box plot of lnIC50 values for 32 different pediatric cancer cell lines following 72-hour treatment with talazoparib. **Supplementary Fig. 2.** A) Dose-response curves following 72-hour treatment with talazoparib in MB (red), ES (orange), OS (purple), RMS (green) and NB (blue) cell lines. All curves represent the average of two replicates and error bares indicate the standard error of the mean (SEM). B) Schematic outlining the high-throughput drug screening approach used in our study. **Supplementary Fig. 3.** Dose response curves of high-throughput screening combining KU- 60019 with the IC15, IC25, and IC50 of talazoparib for each cell line in A673 (orange, ES), D341 (red, MB), HOS (purple, OS), NGP (blue, NB) and RMS-YM (green, RMS). Curves are normalized to talazoparib monotherapy and represent singlicate data. **Supplementary Fig. 4.** Dose response curves of high-throughput screening combining SN-38 with the IC15, IC25, and IC50 of talazoparib for each cell line in A673 (orange, ES), D341 (red, MB), HOS (purple, OS), NGP (blue, NB) and RMS-YM (green, RMS). Curves are normalized to talazoparib monotherapy and represent singlicate data. **Supplementary Fig. 5.** Dose-response curves following 72-hour treatment with talazoparib and SN-38. All curves represent the average of two replicates and error bares indicate the standard error of the mean (SEM). **Supplementary Fig. 6.** Dose-response curves following 72-hour treatment with olaparib and SN-38. All curves represent the average of two replicates and error bares indicate the standard error of the mean (SEM). **Supplementary Fig. 7.** Dose-response curves following 72-hour treatment with pamiparib and SN-38. All curves represent the average of two replicates and error bares indicate the standard error of the mean (SEM). **Supplementary Fig. 8.** Dose-response curves following 72-hour treatment with talazoparib and topotecan. All curves represent the average of two replicates and error bares indicate the standard error of the mean (SEM). **Supplementary Fig. 9.** Dose-response curves following 72-hour treatment with olaparib and topotecan. All curves represent the average of two replicates and error bares indicate the standard error of the mean (SEM). **Supplementary Fig. 10.** Dose-response curves following 72-hour treatment with pamiparib and topotecan. All curves represent the average of two replicates and error bares indicate the standard error of the mean (SEM). **Supplementary Fig. 11.** GSEA showing biological pathways (KEGG) that are positively associated with talazoparib sensitivity in adult cell lines included in the GDSC2 dataset (adjusted *p* value < − 0.001).

## Data Availability

The datasets analyzed during the current study are available at https://www.cancerrxgene.org/. All remaining data generated or analyzed during this study are included in this published article and its supplementary information files.

## Abbreviations

ATM: Ataxia-Telangiectasia Mutated
AUC: Area under the curve
ES: Ewing sarcoma
GDSC2: Genomics of Drug Sensitivity in Cancer
MB: Medulloblastoma
NB: Neuroblastoma
OS: Osteosarcoma
RMS: Rhabdomyosarcoma
