# Supplementary material for: The potential of PARP as a therapeutic target across pediatric solid malignancies
Source: BMC Cancer. 2023 Apr 5;23:310. doi: 10.1186/s12885-022-10319-7 (PMC10077757; doi:10.1186/s12885-022-10319-7)
Supplement: Supplementary file 2 — Additional file 2: Supplementary Fig. 1. Box plot of lnIC50 values for 32 different pediatric cancer cell lines following 72-hour treatment with talazoparib. Supplementary Fig. 2. A) Dose-response curves following 72-hour treatment with talazoparib in MB (red), ES (orange), OS (purple), RMS (green) and NB (blue) cell lines. All curves represent the average of two replicates and error bares indicate the standard error of the mean (SEM). B) Schematic outlining the high-throughput drug screening approach used in our study. Supplementary Fig. 3. Dose response curves of high-throughput screening combining KU- 60019 with the IC15, IC25, and IC50 of talazoparib for each cell line in A673 (orange, ES), D341 (red, MB), HOS (purple, OS), NGP (blue, NB) and RMS-YM (green, RMS). Curves are normalized to talazoparib monotherapy and represent singlicate data. Supplementary Fig. 4. Dose response curves of high-throughput screening combining SN-38 with the IC15, IC25, and IC50 of talazoparib for each cell line in A673 (orange, ES), D341 (red, MB), HOS (purple, OS), NGP (blue, NB) and RMS-YM (green, RMS). Curves are normalized to talazoparib monotherapy and represent singlicate data. Supplementary Fig. 5. Dose-response curves following 72-hour treatment with talazoparib and SN-38. All curves represent the average of two replicates and error bares indicate the standard error of the mean (SEM). Supplementary Fig. 6. Dose-response curves following 72-hour treatment with olaparib and SN-38. All curves represent the average of two replicates and error bares indicate the standard error of the mean (SEM). Supplementary Fig. 7. Dose-response curves following 72-hour treatment with pamiparib and SN-38. All curves represent the average of two replicates and error bares indicate the standard error of the mean (SEM). Supplementary Fig. 8. Dose-response curves following 72-hour treatment with talazoparib and topotecan. All curves represent the average of two replicates and error bares indicate t [file 12885_2022_10319_MOESM2_ESM.pdf]

**Supplementary Data**

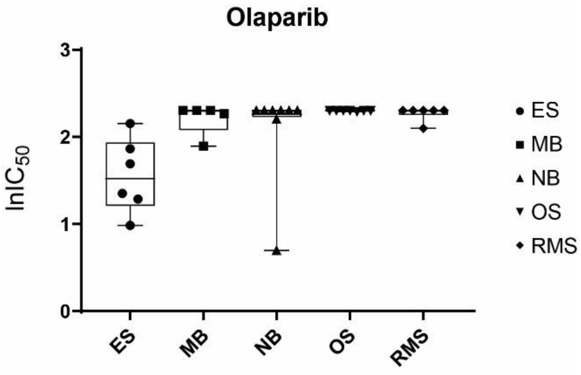


**Supplementary Figure 1-** Box plot of ln$IC_{50}$ values for 32 different pediatric cancer cell lines following 72-hour treatment with talazoparib.

a)

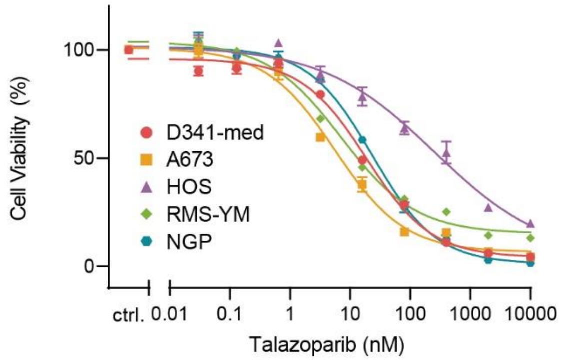


b)

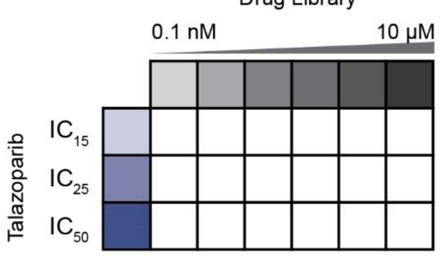


**Supplementary Figure 2- A)** Dose-response curves following 72-hour treatment with talazoparib in MB (red), ES (orange), OS (purple), RMS (green) and NB (blue) cell lines. All curves represent the average of two replicates and error bares indicate the standard error of the mean (SEM). **B)** Schematic outlining the high-throughput drug screening approach used in our study.

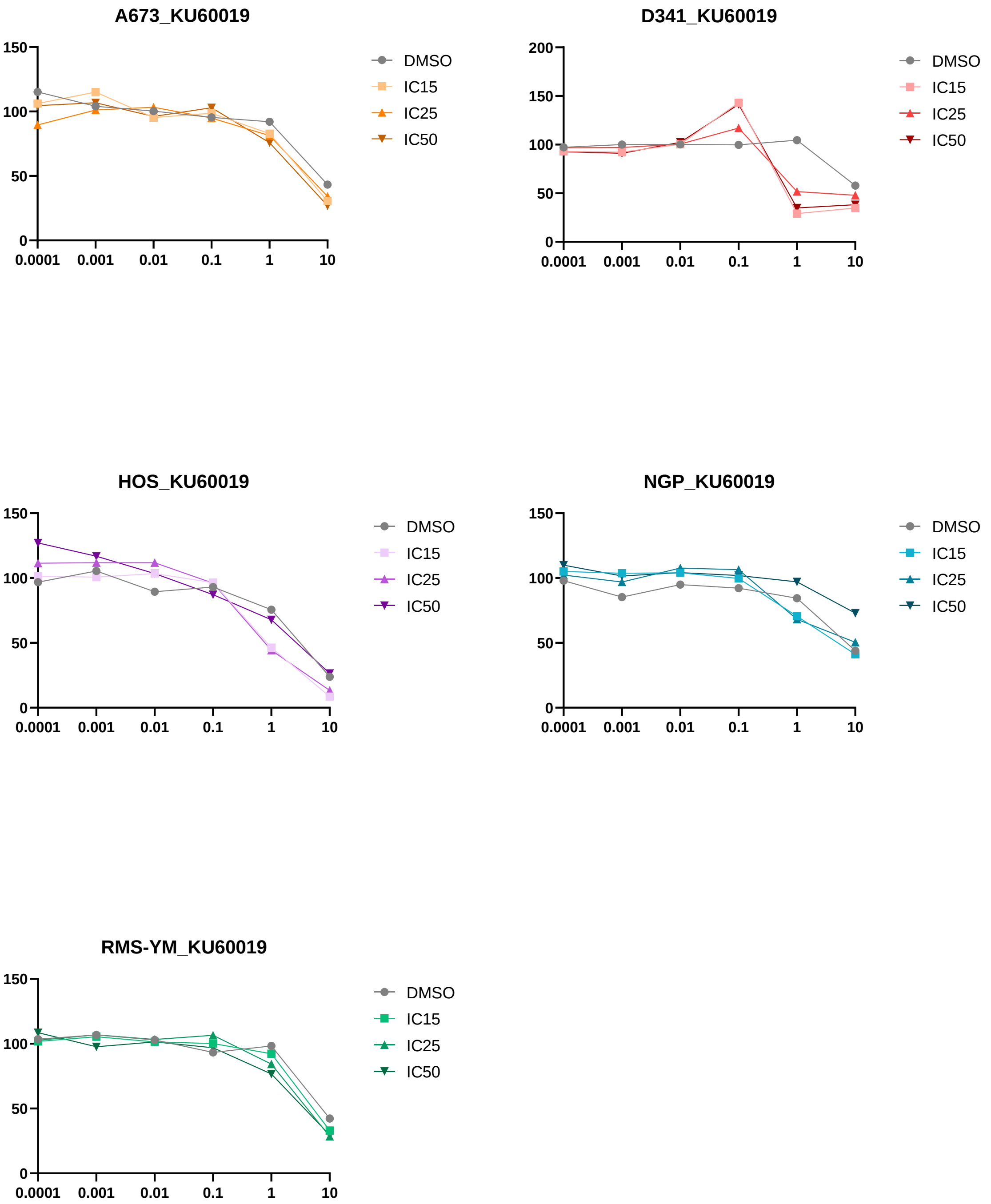


**Supplementary Figure 3-** Dose response curves of high-throughput screening combining KU-60019 with the $IC_{15}$, $IC_{25}$, and $IC_{50}$ of talazoparib for each cell line in A673 (orange, ES), D341 (red, MB), HOS (purple, OS), NGP (blue, NB) and RMS-YM (green, RMS). Curves are normalized to talazoparib monotherapy and represent singlicate data.

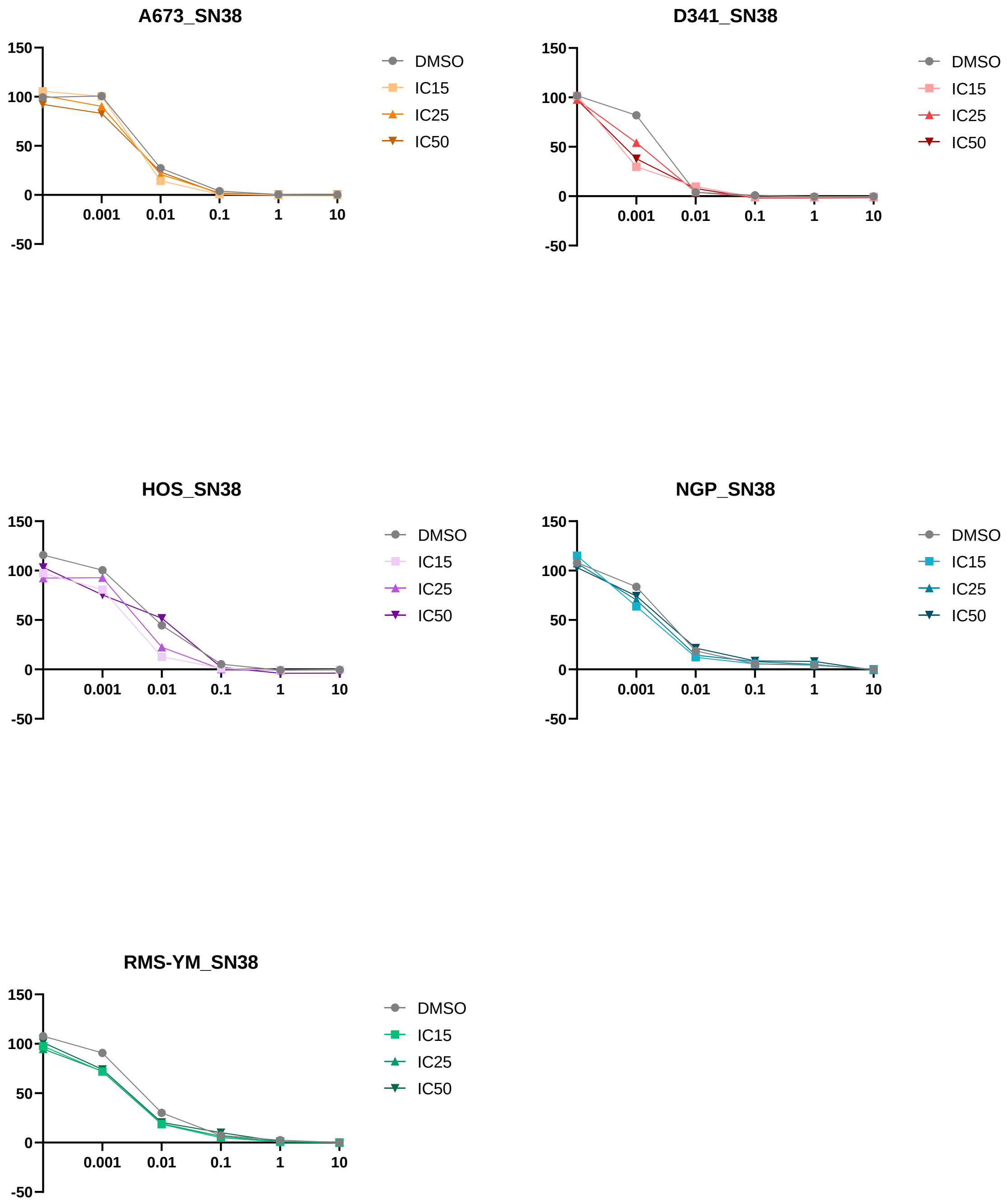


**Supplementary Figure 4-** Dose response curves of high-throughput screening combining SN-38 with the $IC_{15}$, $IC_{25}$, and $IC_{50}$ of talazoparib for each cell line in A673 (orange, ES), D341 (red, MB), HOS (purple, OS), NGP (blue, NB) and RMS-YM (green, RMS). Curves are normalized to talazoparib monotherapy and represent singlicate data.

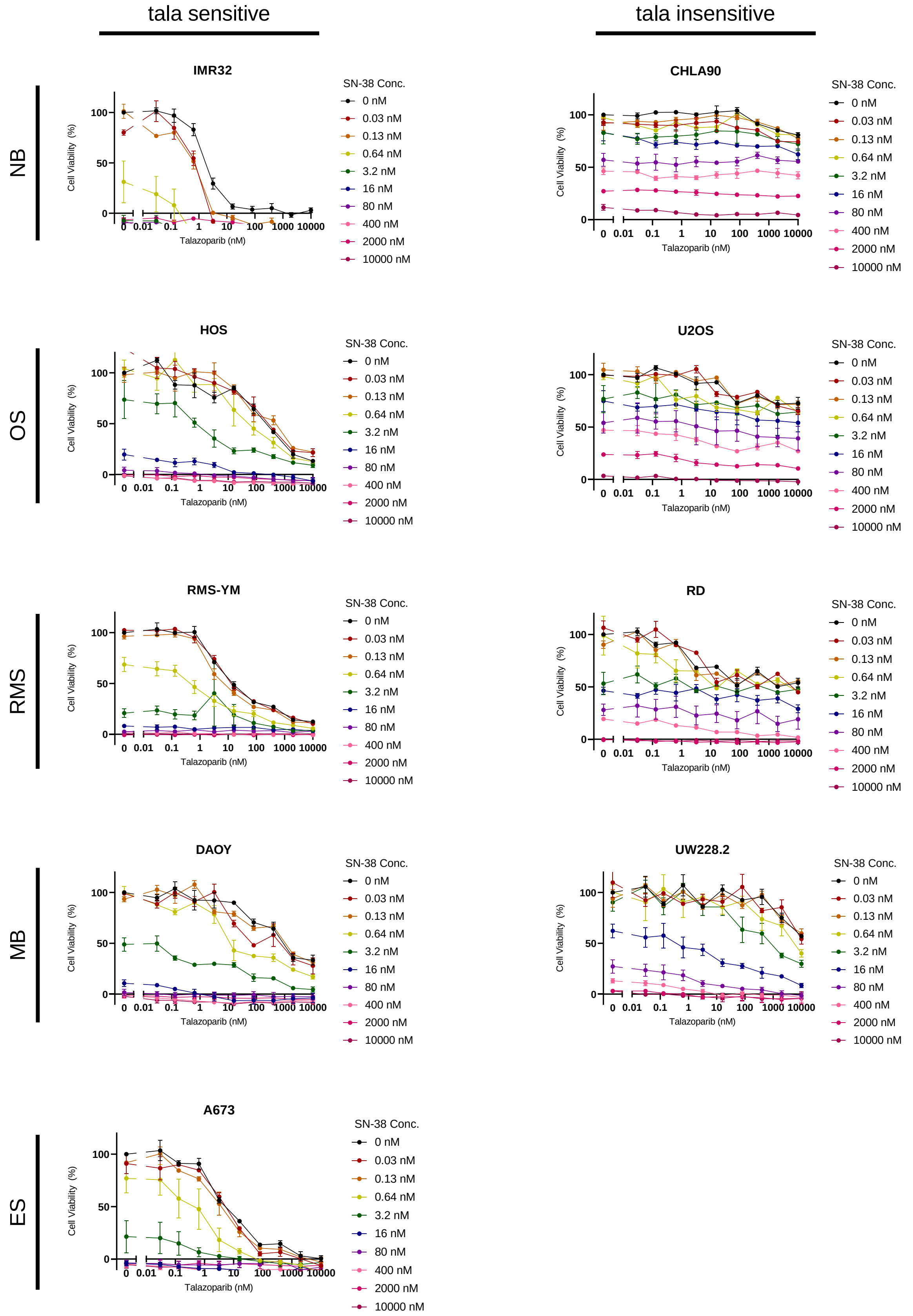


**Supplementary Figure 5-** Dose-response curves following 72-hour treatment with talazoparib and SN-38. All curves represent the average of two replicates and error bares indicate the standard error of the mean (SEM).

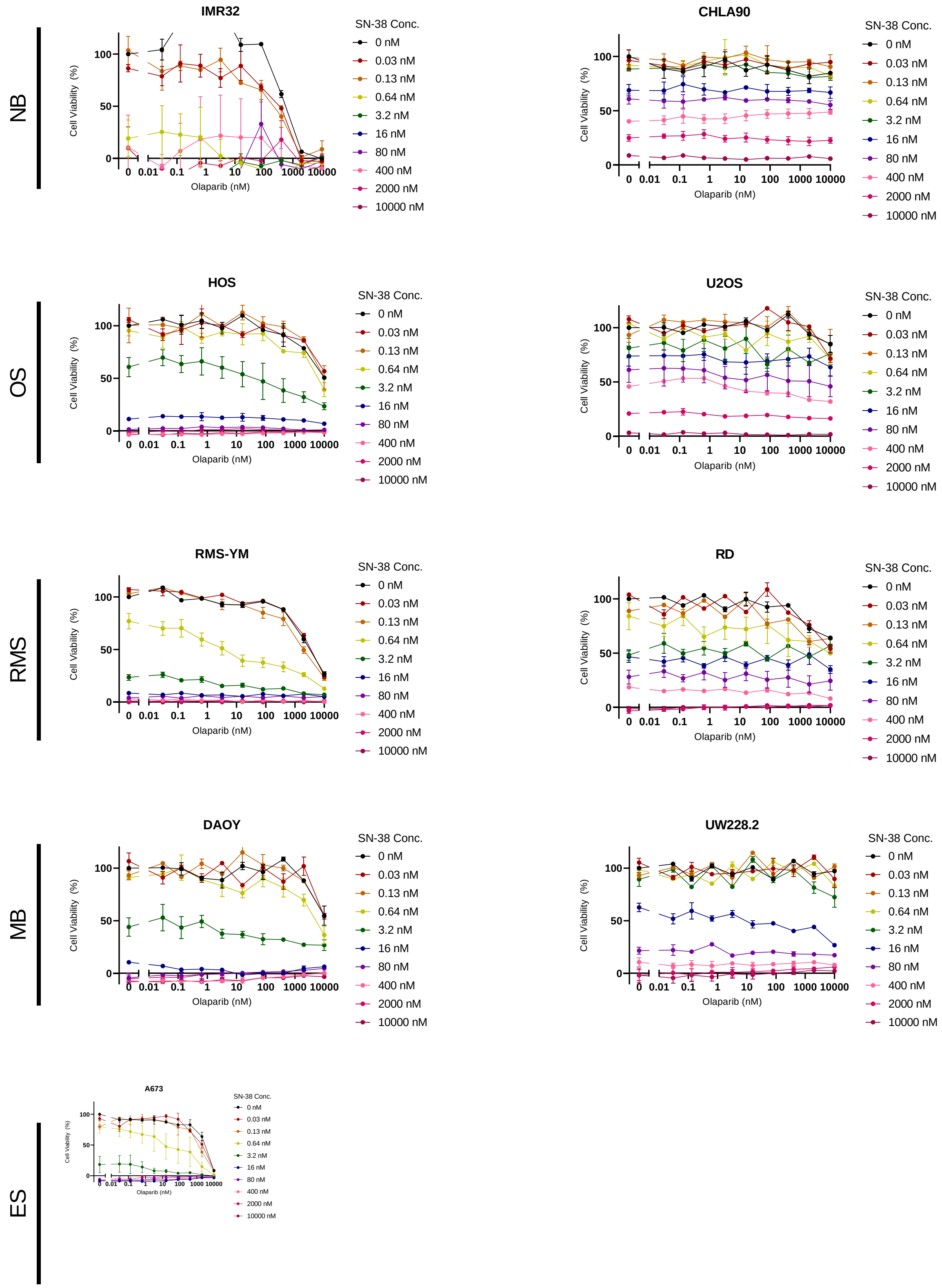


**Supplementary Figure 6-** Dose-response curves following 72-hour treatment with olaparib and SN-38. All curves represent the average of two replicates and error bares indicate the standard error of the mean (SEM).

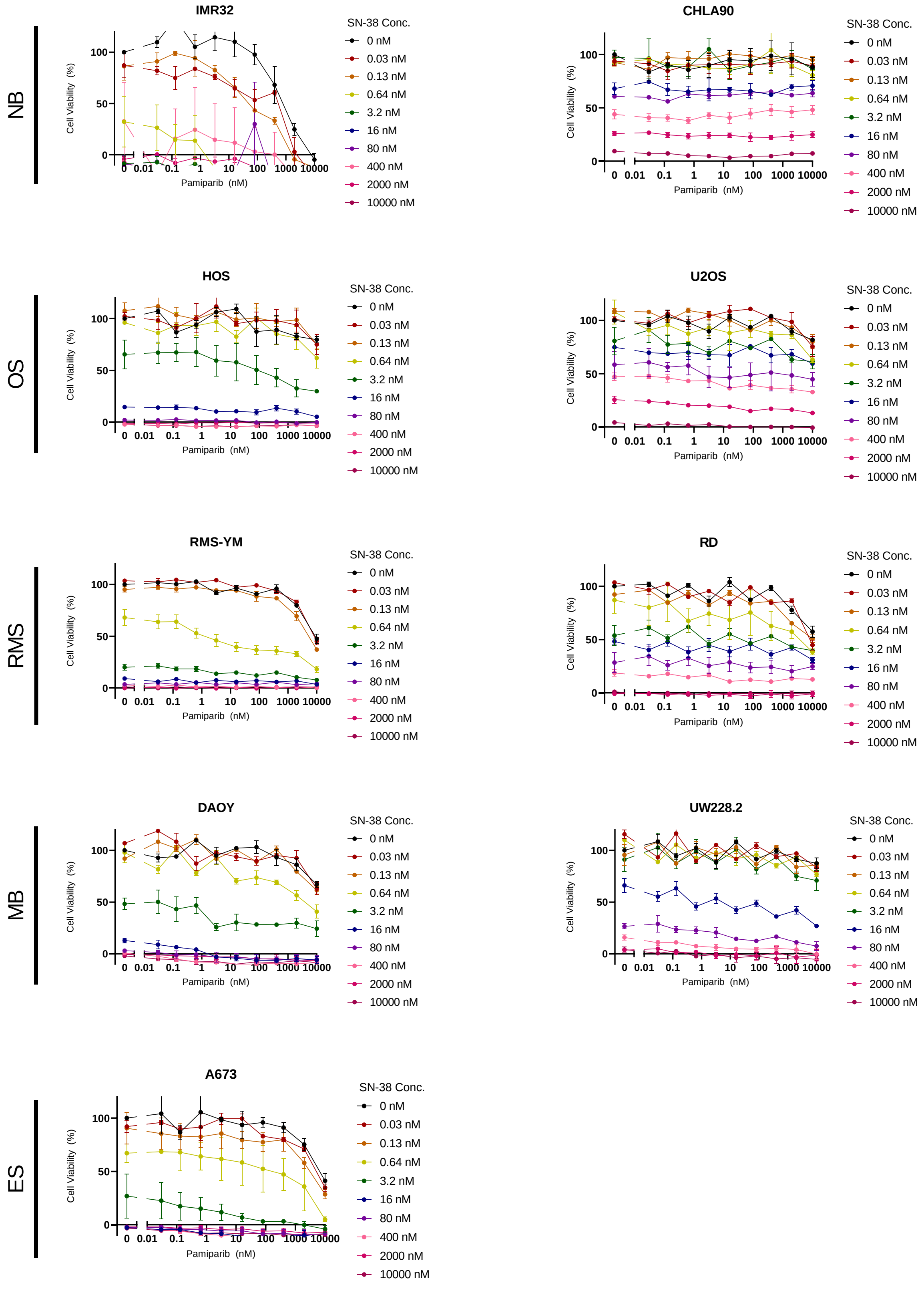


**Supplementary Figure 7-** Dose-response curves following 72-hour treatment with pamiparib and SN-38. All curves represent the average of two replicates and error bares indicate the standard error of the mean (SEM).

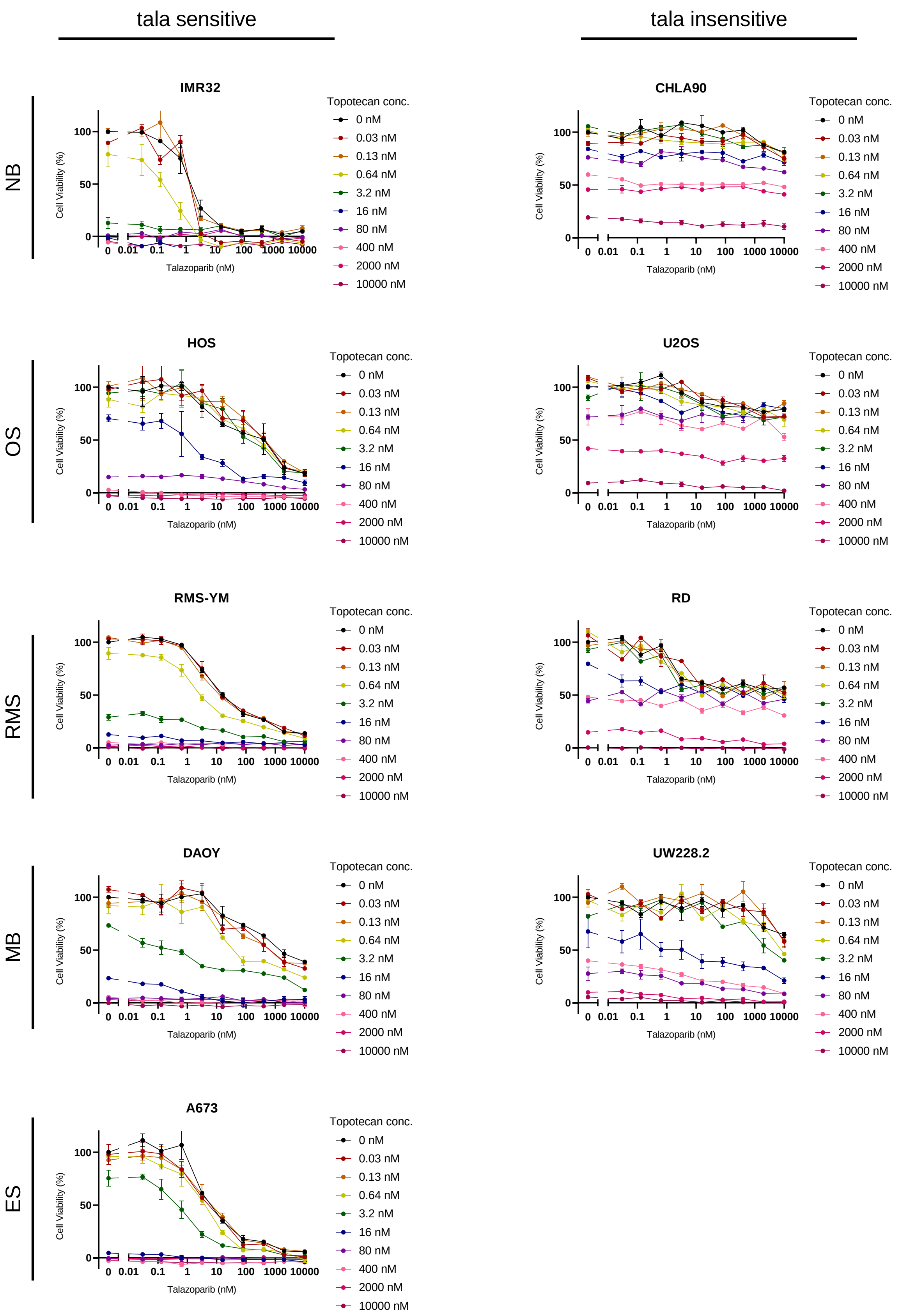


**Supplementary Figure 8-** Dose-response curves following 72-hour treatment with talazoparib and topotecan. All curves represent the average of two replicates and error bares indicate the standard error of the mean (SEM).

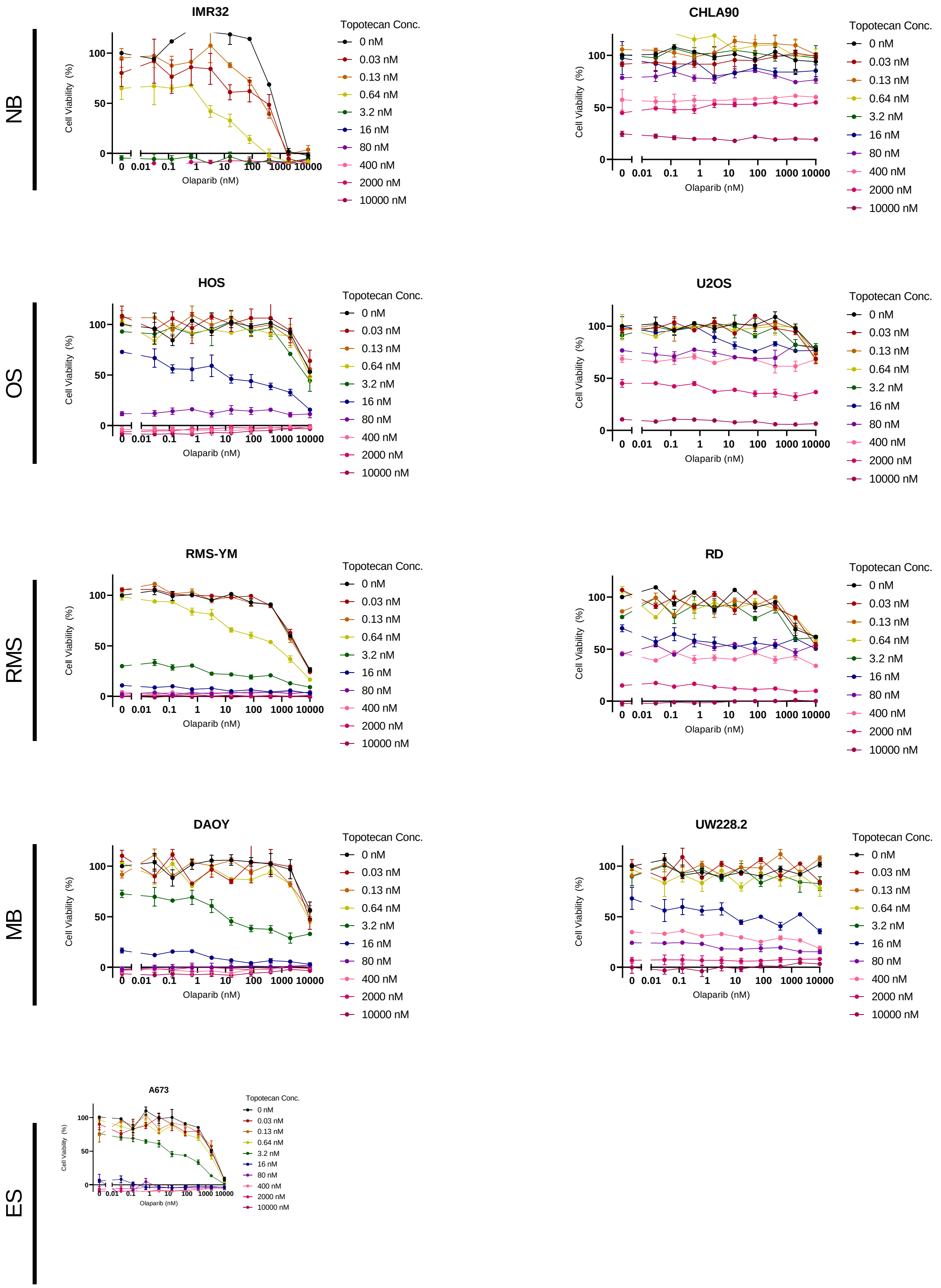


**Supplementary Figure 9-** Dose-response curves following 72-hour treatment with olaparib and topotecan. All curves represent the average of two replicates and error bares indicate the standard error of the mean (SEM).

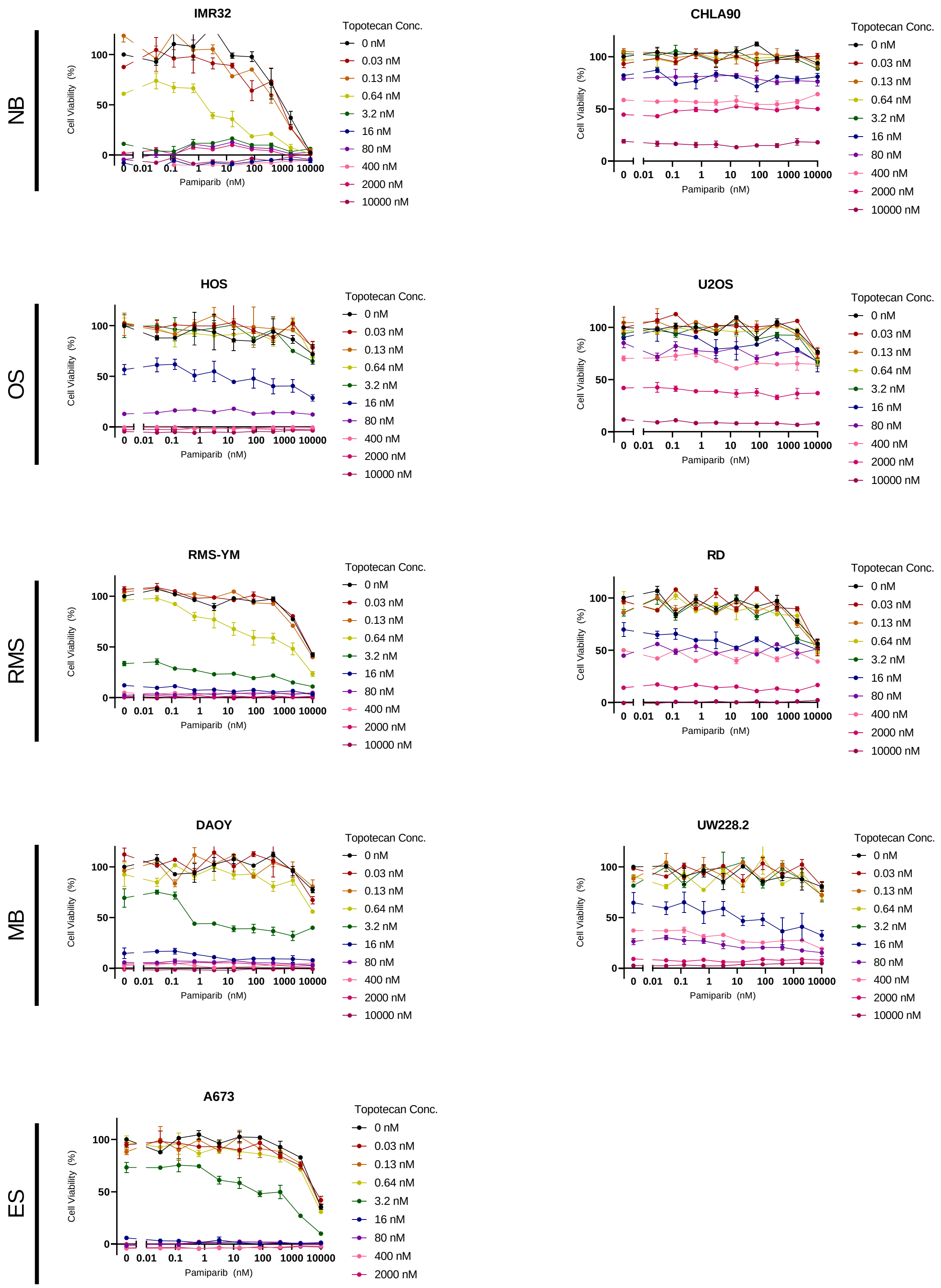


**Supplementary Figure 10-** Dose-response curves following 72-hour treatment with pamiparib and topotecan. All curves represent the average of two replicates and error bares indicate the standard error of the mean (SEM).

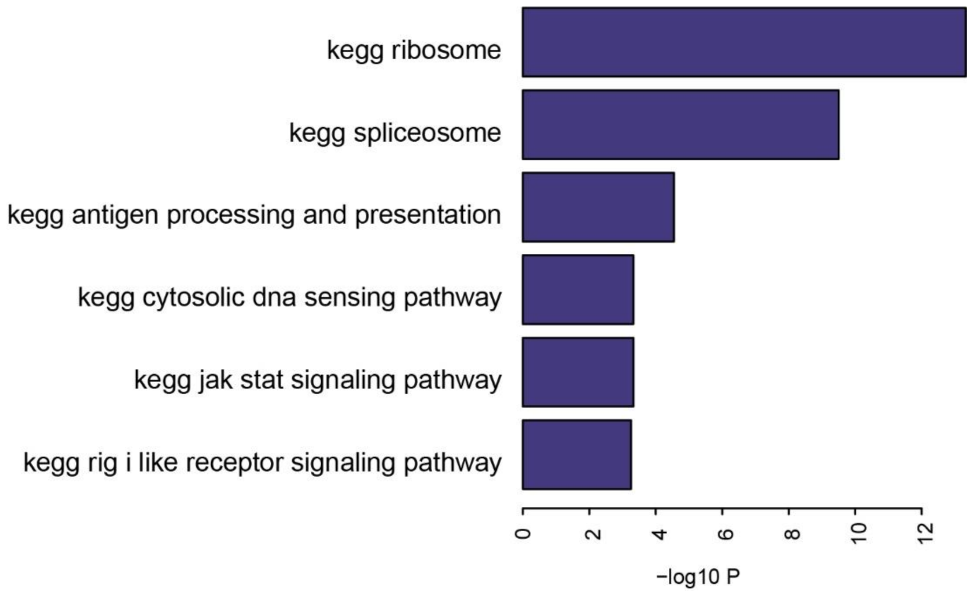


**Supplementary Figure 11-** GSEA showing biological pathways (KEGG) that are positively associated with talazoparib sensitivity in adult cell lines included in the GDSC2 dataset (adjusted p value < -0.001).
